# Ascorbic acid 2-phosphate-releasing poly-l-lactide-co-epsilon-caprolactone membranes enhance tissue regeneration: first *in vivo* insights for pelvic organ prolapse

**DOI:** 10.1093/rb/rbaf097

**Published:** 2025-09-18

**Authors:** Alma Kurki, Markus Hannula, Susanna Miettinen, Henriikka Teittinen, Kaarlo Paakinaho, Jari Hyttinen, Jere Lindén, Reetta Sartoneva

**Affiliations:** Faculty of Medicine and Health Technology (MET), Tampere University, Tampere 33520, Finland; Tays Research Services, Wellbeing Services County of Pirkanmaa, Tampere University Hospital, Tampere 33520, Finland; Faculty of Medicine and Health Technology (MET), Tampere University, Tampere 33520, Finland; Faculty of Medicine and Health Technology (MET), Tampere University, Tampere 33520, Finland; Tays Research Services, Wellbeing Services County of Pirkanmaa, Tampere University Hospital, Tampere 33520, Finland; Biomendex Oy, Tampere 33720, Finland; Faculty of Medicine and Health Technology (MET), Tampere University, Tampere 33520, Finland; Biomendex Oy, Tampere 33720, Finland; Faculty of Medicine and Health Technology (MET), Tampere University, Tampere 33520, Finland; Faculty of Veterinary Medicine, Department of Veterinary Biosciences and Finnish Centre for Laboratory Animal Pathology, HiLIFE, University of Helsinki, Helsinki 00790, Finland; Faculty of Medicine and Health Technology (MET), Tampere University, Tampere 33520, Finland; Tays Research Services, Wellbeing Services County of Pirkanmaa, Tampere University Hospital, Tampere 33520, Finland; Department of Obstetrics and Gynaecology, Wellbeing Services County of South Ostrobothnia, Seinäjoki 60220, Finland

**Keywords:** pelvic organ prolapse, collagen, poly-l-lactide-co-epsilon-caprolactone, ascorbic acid 2-phosphate, *in vivo*

## Abstract

Pelvic organ prolapse (POP) significantly impacts women’s health and quality of life. There is a critical need for alternative biomaterials for surgical POP repair, driven by complications associated with conventional non-absorbable vaginal meshes. As ascorbic acid 2-phosphate (A2P) has been demonstrated to enhance collagen production and cell proliferation *in vitro*, this study investigated absorbable A2P-releasing poly-l-lactide-co-epsilon-caprolactone (PLCL) membranes in the first *in vivo* study to evaluate their potential to promote tissue regeneration for POP treatment. Biomaterials (PLCL, PLCL_4%A2P_, PLCL_8%A2P_ and commercial polypropylene (PP) mesh) were implanted subcutaneously on the abdominal fascia of female Sprague–Dawley rats, and tissue samples were collected for tensile testing and histological analysis at 1-week, 1-month and 6-month time points. Histological samples were analysed using X-ray micro-computed tomography, histological stains, and primary antibodies targeting type I and type III collagen to assess connective tissue regeneration and material degradation. The PLCL_A2P_ groups demonstrated enhanced tissue strength without increased stiffness, compensating for material degradation through tissue regeneration. Moreover, collagen amount was increased in the PLCL_4%A2P_ and PLCL_8%A2P_ groups, without signs of adverse fibrosis. Our results suggest that A2P-releasing PLCL_4%A2P_ and PLCL_8%A2P_ membranes enhance tissue strength and collagen deposition *in vivo*, being a potential alternative for POP repair.

## Graphical Abstract

**Figure:**
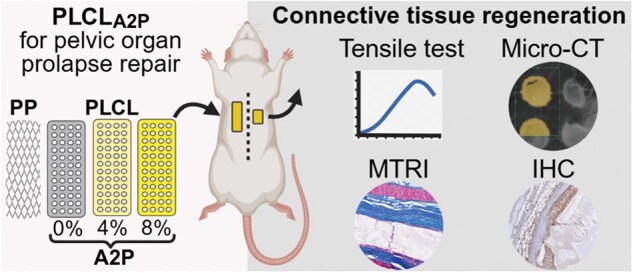

## Introduction

Millions of women globally are affected by pelvic organ prolapse (POP), a common gynaecologic condition where pelvic organs, such as the bladder or uterus, descend into the vagina due to weakened support of pelvic floor ligaments, fascia, and musculature [1]. POP has a multifactorial aetiology, including pregnancy, obesity and ageing. Major symptoms include vaginal bulge, pelvic pressure, and urinary, bowel, and sexual dysfunction, impacting quality of life [1, 2]. Up to 13–18% of women are likely to require surgical repair for POP during their lifetime [1, 3]. Native tissue is typically utilized in the primary repair, but recurrence rates even up to 30% have been reported [4]. Following a failed primary repair, synthetic vaginal meshes, such as polypropylene (PP), have been previously used. Although mesh surgeries may provide better long-term outcomes, complications, such as pelvic pain, infections and tissue erosion, have raised safety concerns [5–7]. Due to the emerging complications and following the FDA-issued high-risk rating in 2019, several countries have restricted or limited their use in POP surgeries [8, 9]. Consequently, there is an evident need for alternative repair material.

POP is associated with disrupted extracellular matrix (ECM) metabolism, leading to unbalanced collagen and elastin content, disorganized fibre architecture, and increased tissue stiffness [10–14]. The ECM, composed primarily of collagen type I (COL I) and III (COL III) and elastin, maintains pelvic tissue stability. COL I provides strength and rigidity, whereas COL III provides elasticity and resilience to stress [15]. Studies have shown reduced total collagen, altered COL III/I ratios and increased collagen proteolysis in POP [12, 14, 16, 17]. Moreover, fibroblasts, the main ECM-producing cells in the pelvic floor, show impaired collagen metabolism at the cellular level, as demonstrated in vaginal fibroblasts derived from POP patients [10, 18]. Therefore, stimulating appropriate collagen production and fibroblast proliferation could be beneficial in pelvic floor reconstructive surgery.

Biodegradable biomaterials offer alternative approaches for POP treatment. For instance, poly-l-lactide-co-epsilon-caprolactone (PLCL) shows potential for POP repair. Its biodegradability, biocompatibility and flexibility make it a promising material for soft tissue engineering applications, and it has been studied for vascular [19–21], neural [22], urethral applications [23, 24] and vaginal and POP applications *in vitro* [25, 26]. To enhance tissue regeneration, biomaterials have been incorporated with various bioactive factors, such as platelet-rich plasma or fibrinogen [27–29], growth factors such as fibroblast growth factor [30–32], and drugs such as oestradiol [33–35]. Despite promising results, the use of growth factors or biological components can complicate clinical application due to regulatory challenges and variable patient responses.

One potential bioactive component for enhancing cell proliferation, collagen and ECM production for POP repair is ascorbic acid 2-phosphate (A2P), a stable derivative of ascorbic acid (AA). AA is a key cofactor in collagen synthesis, and it is essential for proper collagen fibre organization [36]. However, due to its susceptibility to oxidation, the more stable derivative A2P is often used instead [37]. Both AA and A2P have been demonstrated to increase stromal cell proliferation and collagen production *in vitro* [23, 25, 26, 38–41]. Notably, A2P-releasing scaffolds have shown potential in enhancing collagen synthesis *in vitro* [23, 25, 26, 40].

We have previously studied A2P-embedded supercritical CO_2_-foamed PLCL scaffolds for vaginal and urethral tissue engineering applications [23, 26], and more recently, A2P-releasing PLCL membranes (PLCL_A2P_) for POP applications *in vitro*, demonstrating enhanced collagen production, maturation and cell proliferation [25]. However, the *in vivo* effectiveness of these materials has not yet been assessed. This study presents the first *in vivo* evaluation of PLCL_A2P_ membranes in a rat model, aiming to validate previous *in vitro* findings. We aimed to evaluate the effect of PLCL_A2P_ membranes on collagen deposition and connective tissue formation, assessing their potential to strengthen fascial tissues for future application in POP repair. We utilized whole-slide images for quantitative image-based histological assessments and histopathology, and X-ray micro-computed tomography (micro-CT) to quantify tissue ingrowth and biomaterial degradation. The hypothesis is that by promoting connective tissue regeneration through the use of A2P-releasing PLCL_A2P_ membranes, the tissue’s mechanical strength could be improved.

## Materials and methods

### Membrane manufacturing

The investigated materials were fabricated via melt extrusion from raw materials using commercial medical grade PLCL (70L/30CL, Corbion, Purac) and investigation grade L-A2P trisodium salt (≥95%, Mw 322.05 g/mol; Sigma Aldrich, St Louis, MO, USA) using a twin-screw extruder. The PLCL and A2P were combined at weight ratios of 4% A2P to 96% of PLCL (PLCL_4%A2P_), and 8% A2P to 92% of PLCL (PLCL_8%A2P_), respectively. The plain PLCL membranes were manufactured without the addition of A2P.

Prior to extrusion under a nitrogen atmosphere, both raw materials were vacuum dried for 72 h at room temperature (RT). The extrusion temperatures were chosen based on the rheological properties of the polymer and kept as low as possible to minimize the risk of A2P thermal degradation. Cut pieces of the extruded rods were compression moulded into plates with a thickness of 0.4 ± 0.03 mm and laser-cut (Epilog Laser Fusion 75W; Epilog Laser, Golden, CO, USA) into 13 × 18 mm membranes with 35 perforations 1.2 mm in diameter, and 13 × 36 mm membranes with 75 perforations 1.2 mm in diameter. The 1.2-mm diameter was selected based on preliminary *in vivo* tests comparing 1.2- and 1.5-mm perforations, as it provided better tissue integration. The number of perforations was chosen to evenly distribute them across the membrane surface to ensure consistent tissue ingrowth, while still retaining enough material to effectively observe its effects on the surrounding tissue. The membranes were washed with ethanol and vacuum dried for at least 24 h to remove all moisture. All samples were sterilized by gamma-irradiation with a minimum dose of 25 kGy.

### Experimental design

Animal experiments were performed under the ethical approval granted by the Regional State Administrative Agency for Southern Finland (license number ESAVI/26321/2021) and following the 3R principles. In addition, Animal Research: Reporting of in Vivo Experiments (ARRIVE) guidelines were followed. A pilot study with 43 animals was previously conducted to optimize the rat model, study setup, and experimental methods for the final animal study setup. Based on the pilot study findings, the implanted membrane size was minimized to better fit the selected implantation site, operation sterility was increased, and the materials were implanted further from the surgical wound to reduce the risk of infection.

A total of 112 female Sprague–Dawley rats (12 weeks old; Janvier Labs, Le Genest-Saint-Isle, France) were used in this study. The animals were housed in cages with enrichment at GLP-certified preclinical facilities at Tampere University and acclimated for 14 days prior to operation. The animals were randomly assigned to six groups: (i) day 0 control (d0 CTRL), (ii) Sham surgery (SS, control group undergoing operation without material implantation), (iii) nonabsorbable PP mesh (Prolene™ Soft; Ethicon, Raritan, NJ, USA), (iv) PLCL, (v) PLCL_4%A2P_ and (vi) PLCL_8%A2P_. Operations were performed on 14-week-old rats, with a bodyweight of 270–350 g. Animals were euthanized, and samples were collected at 1 week, 1 month and 6 months post-operation, except for d0 CTRL group which was euthanized at the time of surgery (*n* = 7/group/time point).

### Surgical procedure

Prior to operation, animals received 1 mg/kg carprofen (Rimadyl vet; Zoetis, Parsippany, NJ, USA) and 0.3 mg/kg buprenorphine (Vetergesic vet; Ceva, Libourne, France) subcutaneously for analgesia and 5 mg/kg of enrofloxacin (Fenoflox vet; Vet Medic Animal Health, Parola, Finland) for preoperative antibiotics. Animals were anaesthetised with isoflurane inhalation (4% for induction followed by 2–3% for maintenance, Attane vet 1000 mg/g, Vet Medic Animal Health). The surgical procedure was performed under aseptic conditions. Fur was removed from the surgical area on the abdomen. The surgical area was disinfected with 2% chlorhexidine gluconate and 70% isopropyl alcohol (ChloraPrep, BD, Franklin Lakes, NJ, USA) and covered with a sterile surgical drape (Evercare; OneMed, Helsinki, Finland). An approximately 2-cm-long skin incision was made along the midline of the abdomen. Thereafter, the skin was detached from the rat’s abdominal fascia using sharp dissection to create a bilateral tissue pouch for the implants. The implants were placed laterally of the midline and fixed on to the abdominal fascia with simple interrupted sutures (4-0 Ethilon, Ethicon Inc.), after which the incision was closed with a resorbable continuous intracutaneous suture (5-0 Monocryl, Ethicon), cleaned with sterile 0.9% NaCl (B. Braun, Melsungen, Germany) and covered with the wound adhesive (LiquiBand^®^ Optima; Advanced Medical Solutions, Winsford, UK).

After surgery, rats were allowed to awaken in a 37°C recovery room and were then transferred to their cages with continuous access to fresh water and food. Postoperatively, the rats received buprenorphine (Vetergesic vet) 8 h after the previous dose, and carprofen (Rimadyl vet) every 24 h for 2 days. Additional doses of analgesia were given if needed. The rats were followed daily for welfare, drinking, eating, urination and defecation. The surgical area was checked for possible complications. Further, the rats were weighed, and the swelling of the abdominal area was registered on days 3 and 7, and weekly thereafter.

### Sample collection

**Figure 1.**
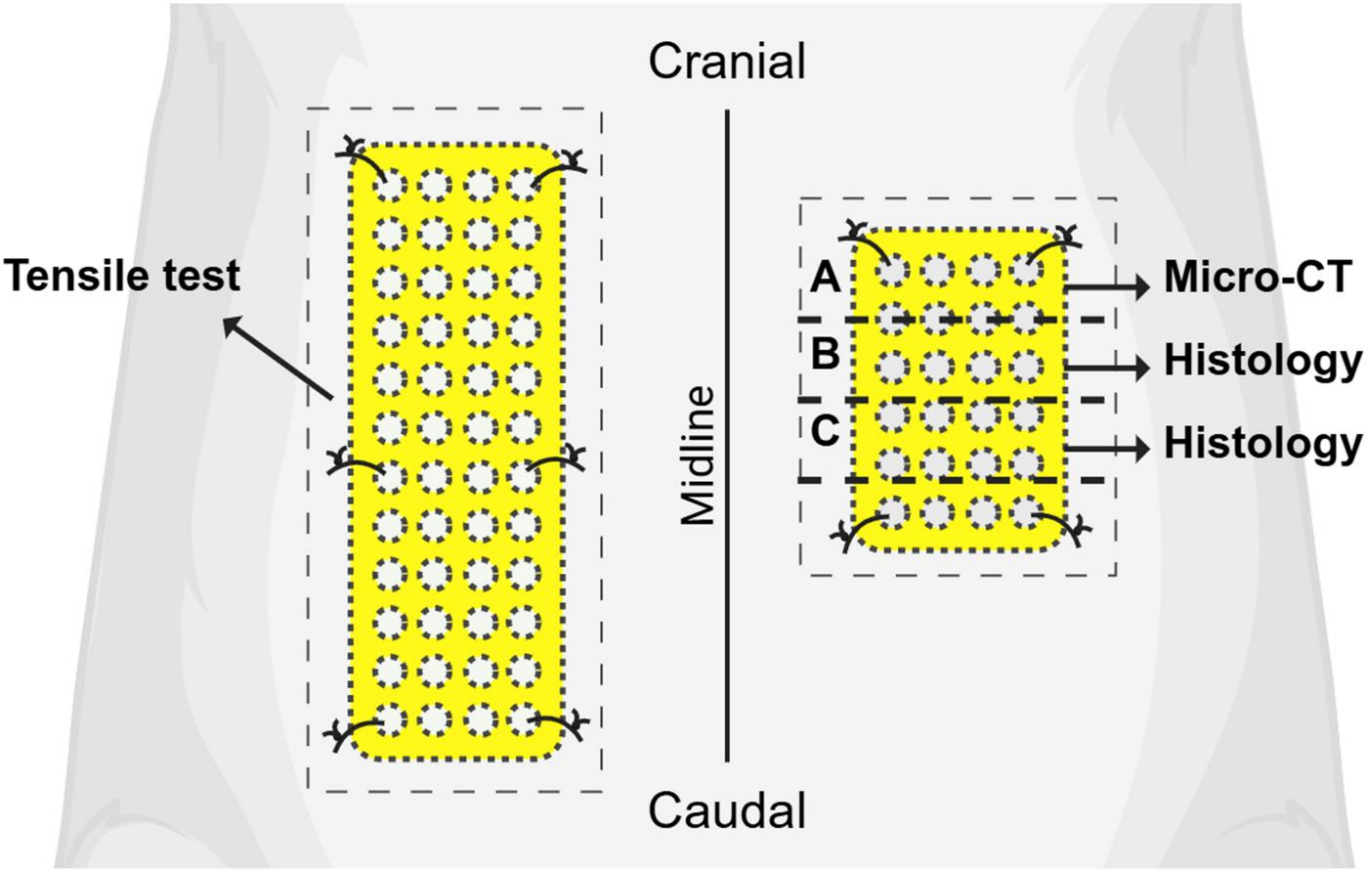
Visualization of sample sectioning for analyses. This figure was created in BioRender by R. Sartoneva (2025) and is available at https://BioRender.com/j36f421.

Animals were euthanized by CO_2_ asphyxiation at day 0, 1 week, 1 month or 6 months. Visualization of the tissue sample processing is presented in Figure 1. Larger material samples were collected for mechanical testing. Skin was carefully removed by sharp dissection, and the samples were stored in 0.9% NaCl at 4°C until uniaxial tensile testing was performed within 48 h of collection. Smaller material samples for histology were collected en bloc and fixed in 10% formalin (Oy Reagena Ltd, Toivala, Finland) at 4°C for 72 h. Formalin-fixed samples were then sectioned: approximately one-quarter was cut cranially for micro-CT imaging (Section A), while the remaining three-quarters were divided into three equal parts and embedded in paraffin. Only sections B and C were used for histological analysis.

### Uniaxial tensile testing of tissue samples

Uniaxial tensile testing was performed using Instron 34TM-5 (Instron, Norwood, MA, USA) to evaluate the biomechanical properties of the tissues. Prior to testing, the collected tissue samples (*n* = 7) were warmed to 37°C in a water bath. The tissue samples, comprising the abdominal wall and the implanted material with skin removed, were cut into rectangular strips approximately 23 mm wide, and their thickness was measured. The sample gauge length was 12 mm and crosshead speed 10 mm/min with 100 N load cell. Initial mechanical properties of the 13 × 36 mm PP, PLCL, PLCL_4%A2P_ and PLCL_8%A2P_ materials were similarly tested prior to implantation. However, their initial cross-sectional area measurements did not account for the filamentous structure of PP or the perforations in PLCL.

From the acquired stress–strain curves, maximum load (N), tensile strength (MPa), strain at maximum load and tensile modulus (MPa) were measured. Maximum load (F_max_) represents the highest applied force before sample rupture. Strain at maximum load was calculated by dividing the displacement at the maximum load (L_Fmax_) by the initial length of the sample (L_o_) (Equation 1). Tensile strength, defined as the maximum stress the sample could withstand before failure, was calculated by dividing F_max_ by the initial cross-sectional area (A_0_) (Equation 2). The elastic modulus, representing the sample stiffness, was determined from the linear portion of the stress–strain curve. However, it is important to note that biological tissues exhibit viscoelastic behaviour rather than the purely linear stress–strain relationship observed in material samples [42].


(1) $$\mathrm{Strain}\;\mathrm{at}\;\mathrm{maximum}\;\mathrm{load}=\;\frac{\left(L_{F_{\max}}-L_{0}\right)}{L_{0}}$$

(2) $$\mathrm{Tensile}\;\mathrm{strength}=\;\frac{F_{\max}}{A_{0}}$$

### Scanning electron microscopy

After tensile testing, material pieces were collected for scanning electron microscopy (SEM) imaging. The samples were sputtered with 5 nm platinum/palladium coating. Samples were imaged with 15 kV acceleration voltage using a JEOL JSM-IT500 SEM microscope (Jeol Ltd Tokyo, Japan).

### X-ray micro-CT analysis

The distribution of A2P-particle volumes in the PLCL_4%A2P_ and PLCL_8%A2P_ membranes was assessed prior to implantation (*n* = 3) using micro-CT. A Zeiss Xradia MicroXCT-400 (Zeiss, Pleasanton, CA, USA) device was used with an 80 kV tube voltage and a 125 µA tube current. A total of 1601 projections were taken with a 4× objective and a 4 s exposure time. The pixel size was 5.64 µm, making the limit of detection 11.24 µm (twice the pixel size). Particle sizes were evaluated using Avizo 2023.1 software (Thermo Fisher Scientific, Waltham, MA, USA). Particles were manually thresholded, and their volumes were measured.

The cranial end of each formalin-fixed PLCL, PLCL_4%A2P_, and PLCL_8%A2P_ tissue samples (Section A in Figure 1) was imaged with micro-CT to quantify the volume of tissue ingrowth into or through membranes and to assess material degradation using 3D image analysis. Fixed tissue samples were dehydrated with an ascending alcohol series and stained with an iodine solution prepared with 1% w/v (10 mg/ml) solid iodine (207772; Sigma Aldrich) in absolute ethanol. Samples were immersed in the iodine solution for 3 days. Thereafter, tissue samples were placed into a syringe to stabilize them during imaging. A 0.4× objective was used with a 19.33 µm pixel size, making the limit of detection 38.55 µm. Other parameters were the same as those used for particle volume analysis. Image stacks were reconstructed in 3D using XMReconstructor software (Zeiss).

Analysis was performed with Dragonfly software version 2024.1 (Comet Technologies Canada Inc., Montreal, Canada). To evaluate different materials and time points, a rectangular cuboid of size 2.15 × 3.34 mm was chosen from each sample. The third dimension varied according to the material height (0.25–0.44 mm). Sizes were defined by imaging pure material samples individually and selecting a cuboid that included 1.5 perforations in the analysis volume. Additionally, a smaller rectangular cuboid (0.6 × 0.6 × 0.25 mm) was selected to further estimate material degradation in material areas without perforations. Tissue sample images were manually thresholded, and soft tissue fractions were measured from the cuboid volumes.

### Histopathology

After 72 h of formalin fixation, B- and C-tissue blocks (Figure 1) were embedded in paraffin and sectioned into 4-µm slices. Separate B and C sections were stained with haematoxylin and eosin (H&E), Masson’s trichrome (MTRI) and Elastin Verhoeff-Van Gieson (EVG) to analyse the structure of connective tissue. Additionally, selected samples from each group were stained with Movat’s pentachrome stain to provide a histological overview of the tissue at 6 months.

For immunohistochemistry, rabbit monoclonal anti-COL I antibody (ab270993; Abcam, Cambridge, UK) and mouse monoclonal anti-COL III (ab6310; Abcam) were used. The tissue sections were first incubated in a PT Module (Lab Vision^TM^; Thermo Fisher Scientific) for 20 min at 99°C in 10 mM citrate buffer, pH 6 (anti-COL I) or 10 mM Tris-EDTA buffer pH 9 (anti-COL III) for antigen retrieval. PBS without detergent was used for the washing steps. Endogenous peroxidase activity was blocked with 3% H_2_O_2_ in PBS for 10 min and nonspecific staining with 20% normal goat serum in PBS at RT for 30 min. Antibody COL I, diluted 1:400 in 1% BSA in PBS, was incubated at RT for 60 min and antibody COL III, diluted 1:300 in 1% BSA in PBS at 4°C overnight. Vectastain ABC-HRP kit (Vector Laboratories, Burlingame, CA, USA) with biotinylated goat anti-rabbit antibody for COL I (1:200, BA-1000; Vector Laboratories, Newark, CA, USA) or biotinylated secondary horse anti-mouse (rat adsorbed) antibody for COL III (1:200, BA-2001;Vector Laboratories) and DAP substrate were used for detection of the primary antibody according to the manufacturer’s instructions. For negative controls, the primary antibodies were replaced with rabbit (1:400, I-1000-5; Vector Laboratories) or mouse (1:300, I-2000-1; Vector Laboratories) control IgGs. Stained sections were scanned using an Olympus VS200 whole-slide scanner (Evident, Tokyo, Japan). All images were blinded to ensure unbiased analysis.

### Visual grading of biomaterial degradation

The degree of material degradation and tissue ingrowth in PLCL, PLCL_4%A2P_ and PLCL_8%A2P_ groups was graded from blinded MTRI-stained B sections using a four-tier scale: 0 = None (no degradation observed), 1 = Minimal (minor cell ingrowth and surface alteration), 2 = Advanced (large tissue ingrowth and cracking) and 3 = Fragmented (material degraded into smaller fragments).

### Visual grading of collagenous capsule formation

Collagenous capsule formation at 6 months in the blinded MTRI-stained B sections was graded based on the amount of dense collagenous fibrous tissue surrounding the implant. Dense collagen was defined as regions of blue-staining parallel collagen strands containing sparse elongated fibrocytes, and their abundance was scored (0 = None, 1 = Minimal, 2 = Moderate, 3 = Marked, 4 = Extensive). The used 0–4 scale was derived from ISO 10993-6:2016 standard but employed the amount of dense collagen to measure fibrous tissue response and capsule formation.

### Quantitative analysis of connective tissue response

**Figure 2.**
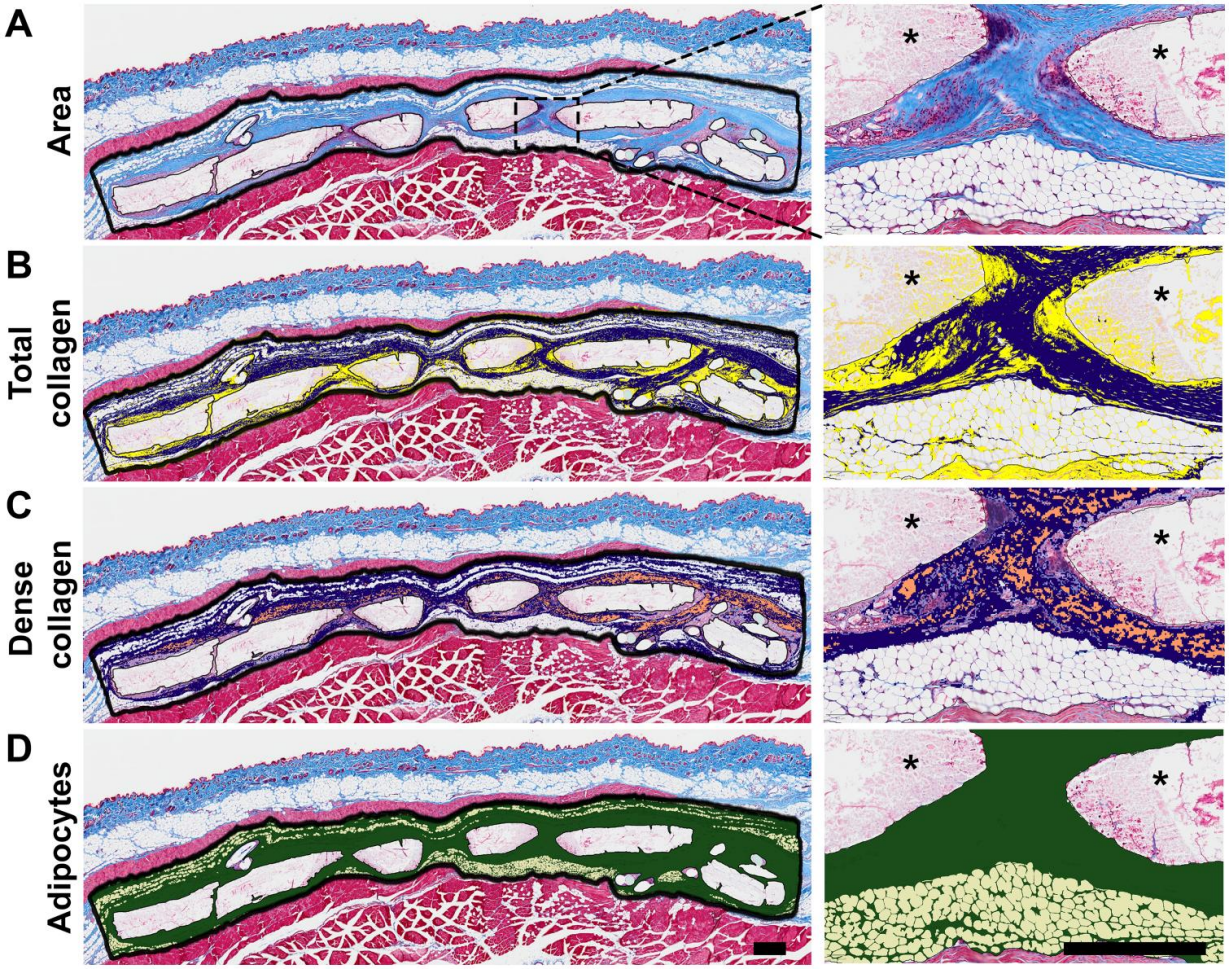
Visualization of the pixel classifiers used to analyse the connective tissue composition within the analysis area. (**A**) Analysis area, (**B**) area of total collagen (dark blue) and other tissue (yellow), (**C**) area of dense collagen (orange) and other collagen (dark blue) and (**D**) area of adipose tissue (light yellow). Scale bar 500 µm. *Biomaterial.

The connective tissue structure at the implantation site in blinded B and C sections was quantitatively analysed at 1 week, 1 month and 6 months with the pixel classification analysis tool of the open-source QuPath software (Ver. 0.5.1) [43]. The analysis area, 11.3 ± 0.5 mm in length, was manually defined using QuPath’s annotation tools to enclose the full thickness of the connective tissue layer between the skin muscle (*panniculus carnosus*) and abdominal muscle layers. Material, mounting stitches or unfocused regions were excluded from the analysis area. The areas of collagen, dense collagen, and adipose tissue and their percentages within the defined analysis area were each quantified with a separately trained pixel classifier. Results are presented as the average of the B and C sections for each rat. An example image of a 6-month PLCL tissue sample, illustrating the defined analysis area and the visual results of the three classifiers, is shown in Figure 2. Detailed descriptions of the pixel classifiers are provided in the Supplementary material (S1).

### Statistical analysis

Quantitative data are presented as the mean ± standard deviation in text and as box-plot graphs in figures. Statistical analysis was performed using IBM SPSS software (version 29.0.1.0; IBM, Chicago, IL, USA). Differences between study groups were evaluated using the Kruskal–Wallis test with Dunn’s pair-wise tests. The *P*-values reported in the results are unadjusted, but they can be adjusted for multiple comparisons using the Bonferroni correction by multiplying the reported *P*-values by 10 (the number of comparisons performed). The d0 CTRL group was excluded from statistical analysis, as their results were used solely to establish the baseline for the study setup. For classification data, statistical differences were evaluated using Fisher–Freeman–Halton exact test. Significance values *P *< 0.05 were considered significant, and *P *< 0.001 as highly significant. The sample size was chosen based on the performed pilot study.

## Results

### The implanted biomaterials

**Figure 3.**
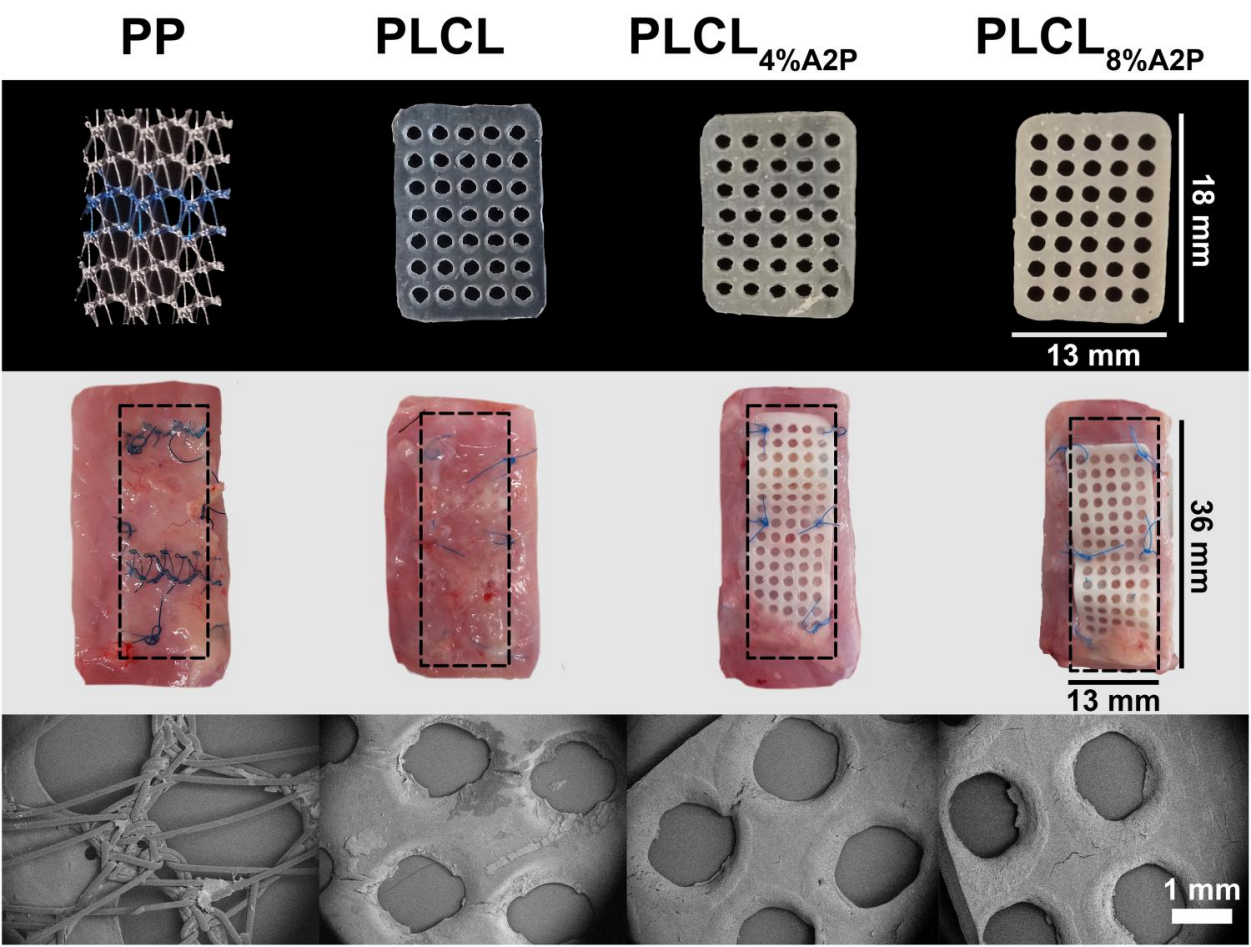
Biomaterials used in this study. Top row shows photographs of the smaller biomaterials used in histology. The middle row shows photographs of the samples collected for the uniaxial tensile test at the 1-week time point. The bottom row shows biomaterials with SEM imaging at the 1-week time point.

**Figure 4.**
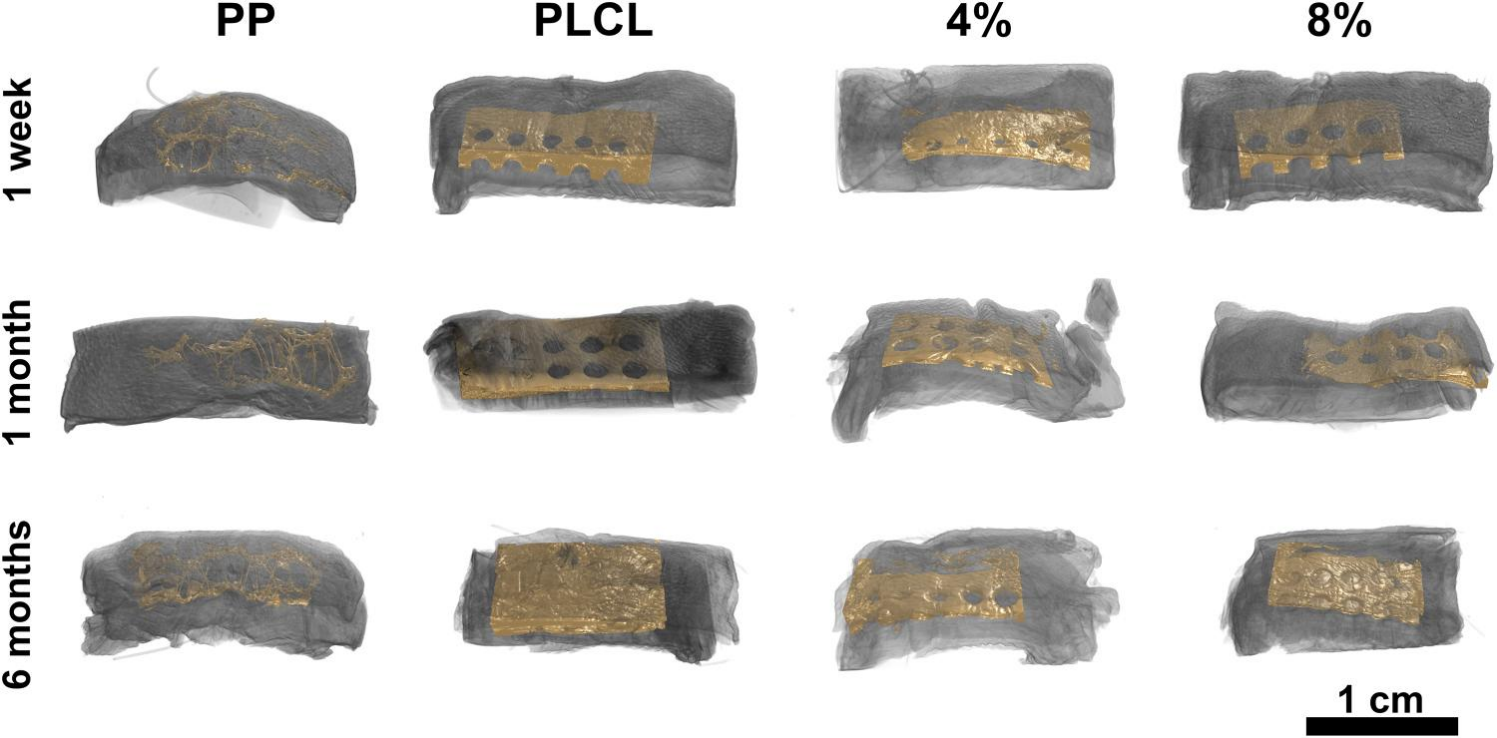
Visualization of PP, PLCL, PLCL_4%A2P_ and PLCL_8%A2P_ materials within tissue as observed with micro-CT imaging.

The outer dimensions of the PP, PLCL, PLCL_4%A2P_, and PLCL_8%A2P_ membranes were the same, yet the perforated PLCL-based membranes were less porous than PP mesh, which also had a smaller contact area with the surrounding tissue (Figure 3). Micro-CT imaging assessed similar A2P particle size distributions in the dry PLCL_4%A2P_ and PLCL_8%A2P_ membranes prior to implantation, considering only particles above the detection limit. Of these measured particles, 60% were under 2000 µm^3^ in volume. Visualization of A2P particles within PLCL_A2P_ membranes is presented in the Supplementary material (S2). Micro-CT images of materials embedded within tissue at 1 week, 1 month and 6 months are presented in Figure 4.

### Animal welfare

The animals exhibited no significant clinical signs during the study, while a total of six animals were excluded from the study due to histologically detected bacterial infections at the operation site: two from the PLCL group, one from the PLCL_4%A2P_ group, and three from the PLCL_8%A2P_ group. In addition, one animal in the SS group developed a respiratory complication during the study period.

Postoperatively, all groups experienced initial weight loss, reaching a minimum at day 3 (SS: −2.8 ± 2.6%; PP −2.6 ± 1.8%; PLCL: −2.9 ± 2.2%; PLCL_4%A2P_: −2.7 ± 2.1%; PLCL_8%A2P_: −2.7 ± 2.5%). Thereafter, weight gain continued without significant differences between groups. By day 14, weight had returned to baseline and was followed by gradual weight gain (Supplementary material S3).

### Implantation site histology

**Figure 5.**
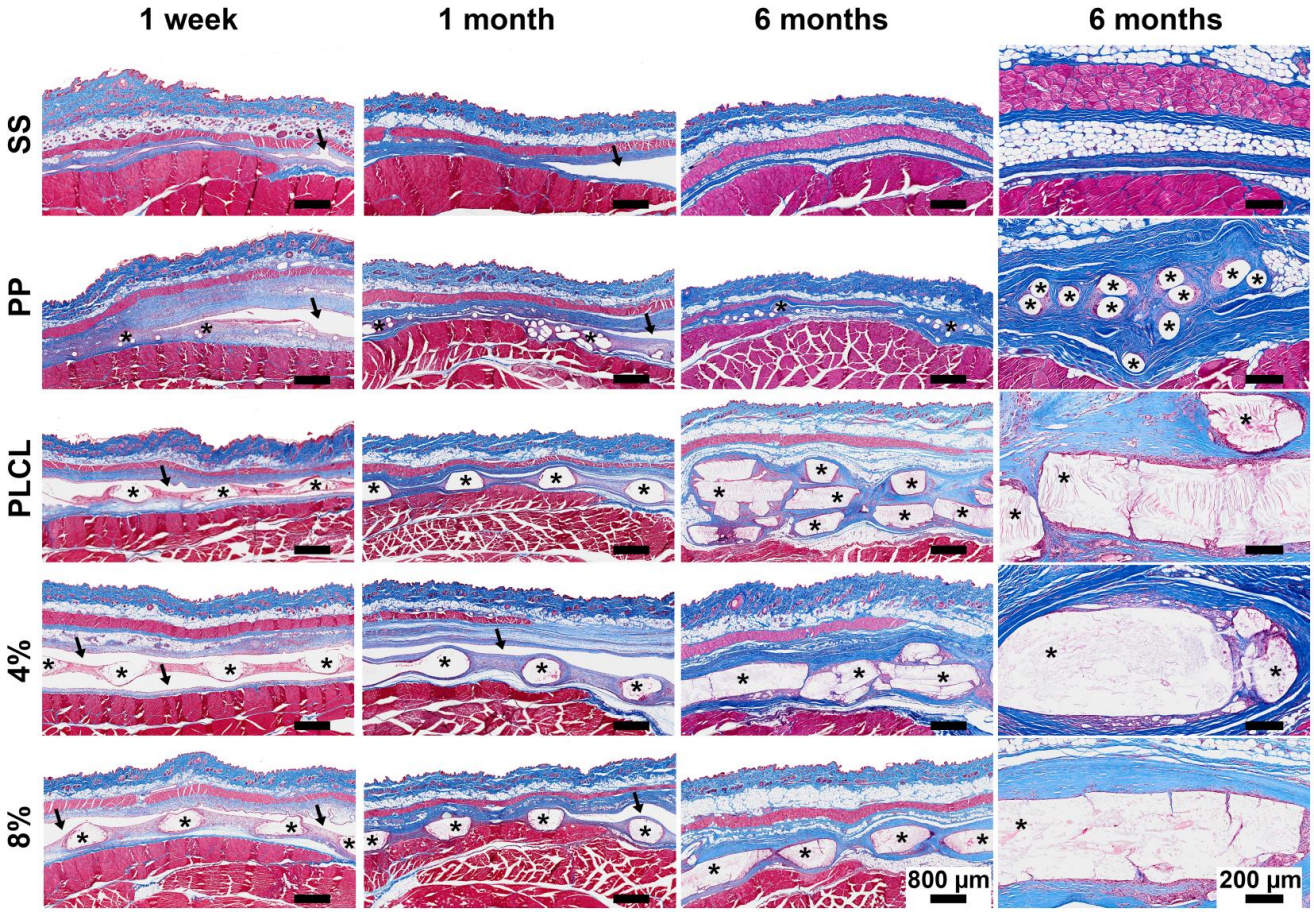
Representative images of tissue samples at 1-week, 1-month and 6-month time points (scale bar 800 µm) with additional close-up images at 6 months (scale bar 200 µm). *Biomaterial; arrows = fluid cavity; 4% = PLCL_4%A2P_, and 8% = PLCL_8%A2P_; PP, polypropylene; SS, sham surgery.

In the H&E- and MTRI-stained histological sections, the PLCL, PLCL_4%A2P_, PLCL_8%A2P_ and PP groups showed generally parallel, though slightly varied, histological changes and progression of implantation site healing, possibly explainable by their difference in pore/material ratio (Figure 5). At 1-week time point, most sections exhibited fluid-filled cavities, variably extending from the cutaneous implant surface to the abdominal surface for material groups. The cavity formation appeared milder in the PP group compared to PLCL, PLCL_4%A2P_ and PLCL_8%A2P_ groups, which showed similar cavity formation between groups, and no histologically detectable material degradation. Immature loose connective tissue with variable amounts of collagen and aggregated fibrin was growing into the holes of PLCL, PLCL_4%A2P_ and PLCL_8%A2P_ membranes. Connective tissue growth on the membrane surfaces was minimal. In the PP, vascularized connective tissue containing variable amounts of collagen was growing through the mesh and promoted tissue integration in non-cavity areas. All SS samples displayed a fluid-filled subcutaneous cavity that appeared to extend through the operation area. A thin layer of macrophages and some multinucleated giant cells generally covered the membranes, whereas in the PP group, a thin zone of mixed macrophages and granulocytes was observed.

By 1 month, the sizes of the fluid-filled cavities had decreased in all material groups, while the PLCL, PLCL_4%A2P_ and PLCL_8%A2P_ groups still showed larger cavities than the PP group. Correspondingly, all PLCL groups exhibited an increased amount of parallel-oriented connective tissue in the membrane holes with generally increased amount of collagen and disappearance of fibrin aggregates. In addition, thin fibroblast tissue strands grew into PLCL, PLCL_4%A2P_ and PLCL_8%A2P_ membranes, with PLCL_4%A2P_ and PLCL_8%A2P_ displaying more pronounced ingrowth than PLCL. In comparison to the 1-week time point, the macrophage and giant cell layer of the PLCL membranes was modestly increased and contained some phagocytized PLCL material, whereas the reaction towards the PP material was reduced. All SS samples still showed variable-sized fluid-filled cavities between the skin and the abdominal muscle.

By 6 months, all materials had integrated into the surrounding tissue. All groups exhibited moderate to marked, likely age-related, increase in adipose tissue in the operation area. The PLCL, PLCL_4%A2P_ and PLCL_8%A2P_ membranes had generally degraded into fragments that were further dissected by fibrous connective tissue and macrophage strands. Dense collagenous capsules (85–260 µm thick), composed of parallel collagen strands with elongated fibrocytes, surrounded the PLCL-based membranes. In comparison, the PP group exhibited similar collagen capsule formation throughout the material; however, it contained some unorganized fibrotic collagen-dense areas with single discernible fibrocytes. A moderate layer of PLCL material-containing macrophages and some multinucleated giant cells generally covered all material surfaces, particularly in areas with advanced material degradation in the PLCL, PLCL_4%A2P_ and PLCL_8%A2P_ groups. Correspondingly, a thin layer of foamy macrophages, single small giant cells and granulocytes covered the PP mesh strands.

**Figure 6.**
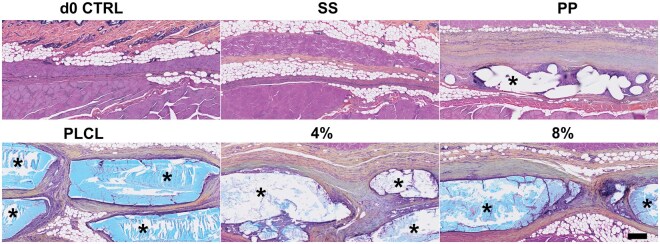
Movat’s pentachrome staining of tissue samples at the 6-month time point. *Biomaterial. Scale bar 200 µm. 4% = PLCL_4%A2P_; 8% = PLCL_8%A2P_; PP, polypropylene; SS, sham surgery.

Movat’s pentachrome staining provided an overview of the tissue response to the implanted biomaterials at the 6-month time point (Figure 6). Collagen deposition (yellow) surrounded PLCL, PLCL_4%A2P_ and PLCL_8%A2P_ membranes and PP mesh. In areas of dense collagen, a slight shade of blue was observed. Interestingly, in the d0 CTRL and SS groups, collagen of the apparently pre-existing connective tissue stained red, similar to muscle tissues. No elastin fibres (black) were detected in the newly formed connective tissue surrounding the materials. Similarly, in the EVG-stained tissue sections, likely pre-existing mature elastin fibres were consistently observed adjacent to muscle layers in all material groups. However, no elastin fibres were detected around the implanted materials, and therefore no quantitative measurements were performed.

### *In vivo* degradation analysis using micro-CT and histology

**Figure 7.**
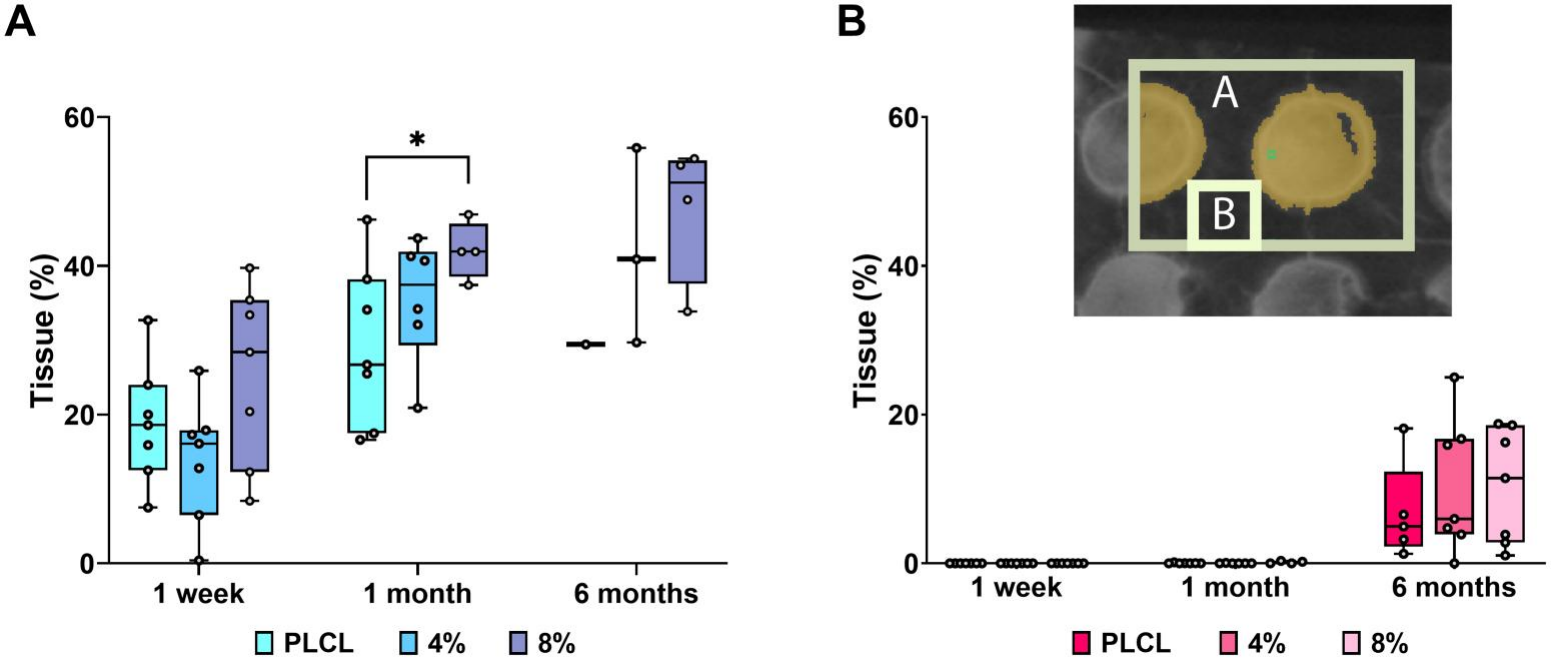
Percentage of tissue ingrowth in PLCL, PLCL_4%A2P_ and PLCL_8%A2P_ membranes. (**A**) Larger sample volume that includes 1.5 perforations in membrane (*n* = 1–7), (**B**) smaller sample volume within the material excluding membrane perforations (*n* = 5–7). The upper-right corner shows a top-view visualization of the measurement volumes. *P < 0.05.

The volume of tissue ingrowth was measured with micro-CT imaging, representing the degradation of the PLCL, PLCL_4%A2P_ and PLCL_8%A2P_ membranes. The percentage of tissue within the measurement volume appeared to increase with higher A2P concentration in both the larger measurement volume, which included 1.5 membrane perforations (Figure 7A), and the smaller measurement volume within material without perforations (Figure 7B). At 1 week and 1 month, more tissue was measured growing through the holes in PLCL_8%A2P_, compared to other membranes. No tissue ingrowth into materials was measured on 1 week or 1 month; however, it is important to note the 40-µm detection limit of the used micro-CT analysis method. The measurement was challenging at the 6-month time point as corresponding areas for the larger measurement volume could not be achieved in all samples due to material degradation, migration, or imperfect sample preparation. As a result, only one PLCL, three PLCL_4%A2P_ and four PLCL_8%A2P_ samples could be measured for the larger volume. Still, the available data suggested an increasing tissue integration, particularly in PLCL_4%A2P_ and PLCL_8%A2P_ samples. All samples could be analysed for the smaller measurement volume. At 6 months, ingrown tissue into the degrading materials was measured for all membranes, with no significant differences.

To support the micro-CT analysis, the degree of material degradation of the bioabsorbable PLCL, PLCL_4%A2P_ and PLCL_8%A2P_ was graded from the blinded MTRI-stained B sections to assess also the thinner tissue strands growing into the materials that were otherwise undetected due to the micro-CT’s limit of detection (Table 1). Material degradation appeared more advanced with an increased amount of A2P, and PLCL_8%A2P_ group showed further degradation at 1-week and 1-month time points (*P *< 0.01). By 6 months, all groups showed a similar degree of degradation.

**Table 1.** Grading of PLCL, PLCL_4%A2P_ and PLCL_8%A2P_ membrane degradation at time points in blinded masson trichrome-stained tissue sections (*N* = 4–7)

Grading of material degradation, *N* (%)					
		PLCL	4%	8%	*P*-value
1 week					<0.001
	None	7 (100)	2 (29)	0	
	Minimal	0	4 (57)	6 (86)	
	Advanced	0	1 (14)	1 (14)	
	Fragmented	0	0	0	
Total (*N*)		7	7	7	
1 month					0.003
	None	7 (100)	2 (33)	0	
	Minimal	0	4 (67)	3 (75)	
	Advanced	0	0	1 (25)	
	Fragmented	0	0	0	
Total (*N*)		7	6	4	
6 months					ns
	None	0	0	0	
	Minimal	0	0	0	
	Advanced	0	1 (14)	1 (14)	
	Fragmented	5 (100)	6 (86)	6 (86)	
Total (*N*)		5	7	7	

4%, PLCL_4%A2P_; 8%, PLCL_8%A2P_.

### A2P enhanced tissue strength without increased stiffness

Uniaxial tensile testing for dry PP, PLCL, PLCL_4%A2P_ and PLCL_8%A2P_ materials (*n* = 3) was performed to assess the material tensile properties prior to *in vivo* implantation (Table 2). In this study, the PP functions as a commercially available reference for the PLCL membranes.

**Table 2.** Tensile properties of PP, PLCL, PLCL_4%A2P_ and PLCL_8%A2P_ biomaterials prior to implantation (mean ± SD) (*N* = 3)

Material tensile properties prior to implantation				
	Max load (N)	Tensile strength (MPa)	Strain at max load	Modulus (MPa)
PP	42.6 ± 1.0	9.0 ± 0.2	0.7 ± 1.2	15.7 ± 1.2
PLCL	11.8 ± 0.7	2.3 ± 0.1	2.0 ± 0.2	15.6 ± 0.1
PLCL_4%A2P_	22.6 ± 1.4	4.3 ± 0.3	2.0 ± 0.1	17.7 ± 2.0
PLCL_8%A2P_	20.4 ± 2.7	4.0 ± 0.5	1.8 ± 0.2	27.5 ± 4.9

**Figure 8.**
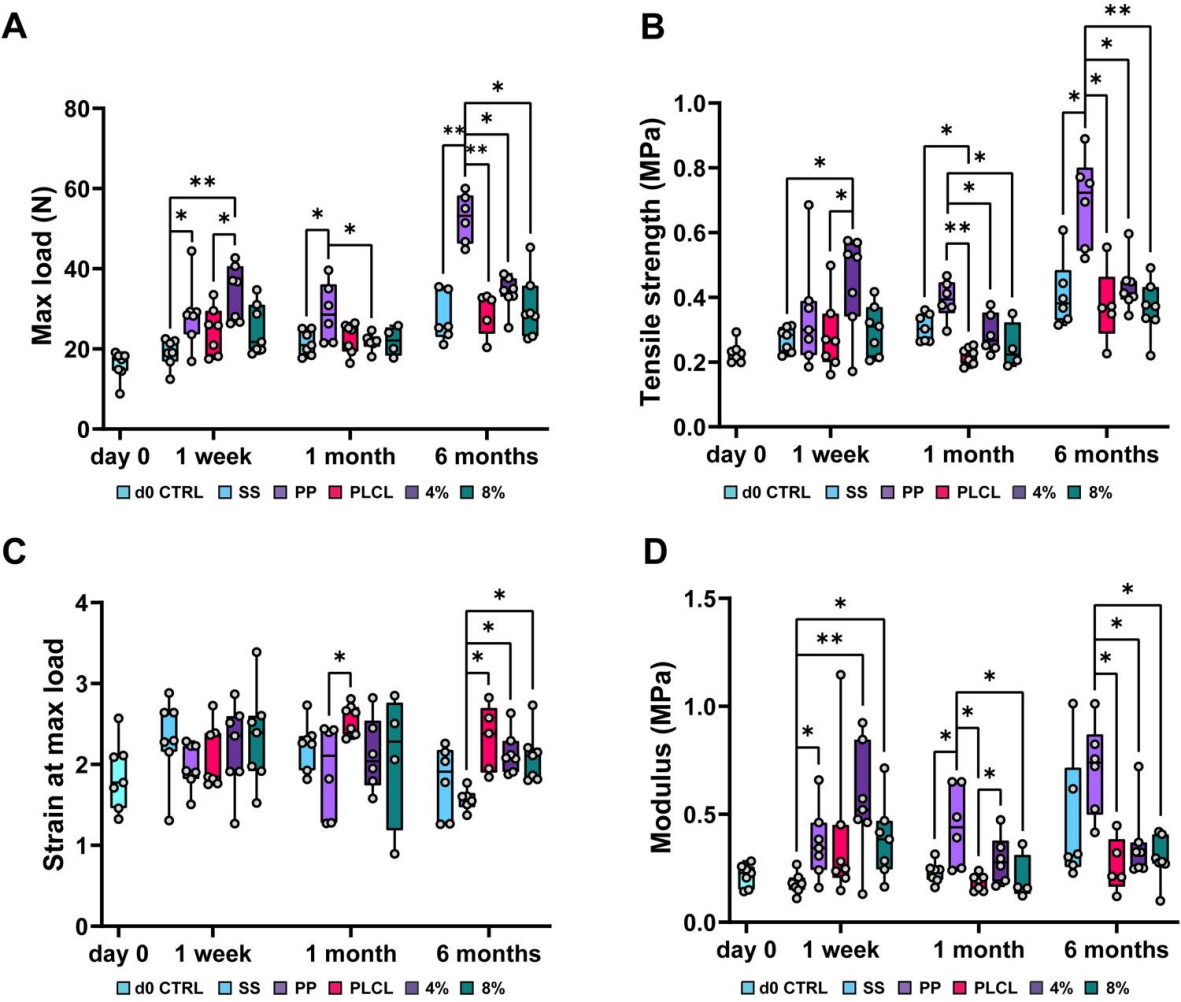
Biomechanical properties of the tissue samples. (**A**) Maximum load (N), (**B**) tensile strength (MPa), (**C**) strain at max load and (**D**) modulus (MPa) at the time points. *N* = 4–7. *P < 0.05, and **P < 0.001. 4%, PLCL_4%A2P_; 8%, PLCL_8%A2P_; PP, polypropylene; SS, sham surgery.

For tissue samples, uniaxial tensile testing was performed on explanted tissue-material samples with skin carefully removed, to assess the material effects on tissue mechanical properties (Figure 8). At 1 week, all materials were still present, strongly influencing the tensile results. By 1 month, degradation of the PLCL, PLCL_4%A2P_ and PLCL_8%A2P_ membranes was observed during sample handling, and by 6 months, they exhibited complete loss of mechanical properties.

The PLCL_4%A2P_ group had the highest maximum load (34 ± 6.4 N) at 1 week, which was significantly higher than the PLCL (24.5 ± 5.5 N; *P *= 0.039) and SS (19 ± 3.3 N; *P *< 0.001) groups. At 1 month, PLCL, PLCL_4%A2P_ and PLCL_8%A2P_ groups exhibited similar maximum load (22.7 ± 3.6 N, 21.7 ± 2 N and 21.9 ± 3.2 N, respectively), yet the PP group had the highest maximum load (29.1 ± 6.7 N). By 6 months, the PLCL_4%A2P_ group had a slightly higher yet not statistically significant maximum load (33.9 ± 4 N) than PLCL (29.1 ± 4.9 N), PLCL_8%A2P_ (30.4 ± 7.3 N) and SS (27.7 ± 5.5 N) groups. The PP group maintained the highest maximum load (52.6 ± 5.5 N; *P *< 0.05). All PLCL-based groups showed a statistically significant decrease in maximum load between 1 week and 1 month due to material degradation, but by 6 months, the values increased to a similar level as the 1-week time point.

The PLCL_4%A2P_ group exhibited the highest tensile strength (0.45 ± 0.14 MPa) at 1 week, significantly to PLCL (0.28 ± 0.1 MPa; *P *= 0.019) and SS groups (0.27 ± 0.04 MPa; *P *= 0.032). At 1 month, PLCL, PLCL_4%A2P_ and PLCL_8%A2P_ groups showed similar tensile strength with no significant differences (0.22 ± 0.02 MPa, 0.29 ± 0.06 MPa and 0.25 ± 0.06 MPa, respectively). The PP group had the highest tensile strength (0.39 ± 0.06 MPa), being significantly higher than PLCL-based groups (*P *< 0.05). At 6 months, PLCL-based groups exhibited similar tensile strength with no statistical differences (PLCL 0.37 ± 0.11 MPa, PLCL_4%A2P_ 0.44 ± 0.07 MPa and PLCL_8%A2P_ 0.37 ± 0.08 MPa), and the PP group had the highest tensile strength (0.7 ± 0.13 MPa) compared to other groups (*P* < 0.05).

No statistical differences in strain were detected at the 1-week time point. At 1 month, the PLCL group had the highest strain at maximum load (2.6 ± 0.2), significantly higher only to the PP group (1.9 ± 0.5; *P *= 0.028). At 6 months, all PLCL-based groups exhibited higher strain (PLCL 2.3 ± 0.4, PLCL_4%A2P_ 2.1 ± 0.2 and PLCL_8%A2P_ 2.1 ± 0.3) compared to the PP group (1.6 ± 0.1; *P *< 0.05). Strain remained similar from 1 week to 6 months for the PLCL, PLCL_4%A2P_ and PLCL_8%A2P_ groups, while the SS and PP groups showed decreased strain.

Modulus at 1 week was lowest in the SS group (0.18 ± 0.05 MPa) compared to material groups: PP 0.37 ± 0.15 MPa (*P *= 0.039), PLCL_4%A2P_ 0.56 ± 0.24 MPa (*P *< 0.001) and PLCL_8%A2P_ 0.38 ± 0.17 MPa (*P *= 0.022). At 1 month, the PLCL_4%A2P_ group had a higher modulus (0.29 ± 0.1 MPa) than the PLCL group (0.19 ± 0.03 MPa; *P *= 0.049). However, the PP group had the highest modulus (0.45 ± 0.17 MPa), being significantly higher than the SS (0.23 ± 0.05 MPa; *P *= 0.048), PLCL (*P *= 0.001) and PLCL_8%A2P_ (0.2 ± 0.09 MPa; *P *= 0.006) groups. At 6 months, modulus was significantly lower in the PLCL-based groups (PLCL 0.26 ± 0.11 MPa, PLCL_4%A2P_ 0.36 ± 0.16 MPa and PLCL_8%A2P_ 0.29 ± 0.1 MPa; *P *< 0.05) compared to the PP group (0.71 ± 0.19 MPa).

### PLCL_A2P_ promoted collagen deposition in tissue

**Figure 9.**
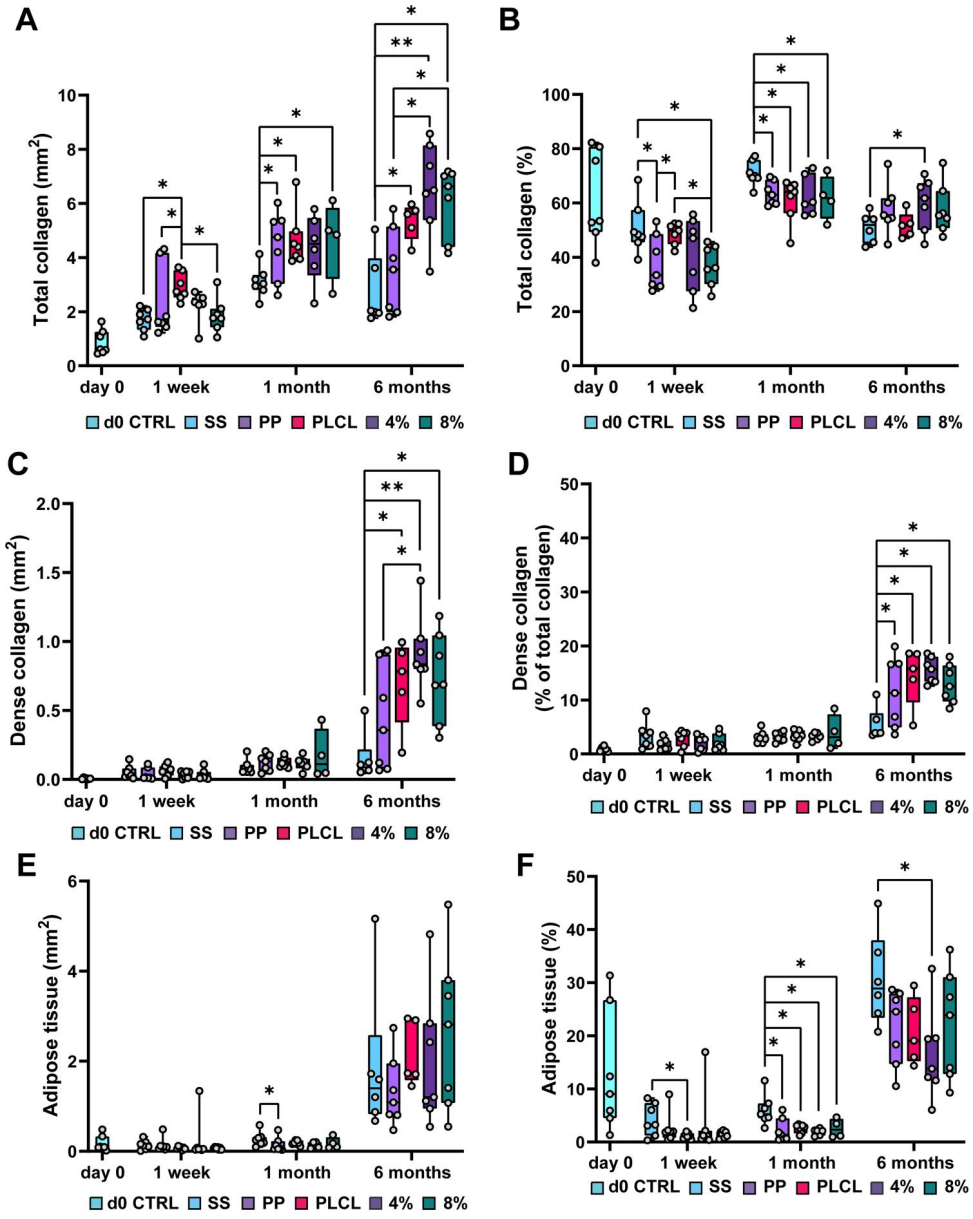
Results of MTRI pixel classifications. (**A**) Total collagen area, (**B**) total collagen percentage, (**C**) dense collagen area, (**D**) percentage of dense collagen within total collagen, (**E**) adipose tissue area and (**F**) percentage of adipose tissue. Note: *y*-axis range differs between subfigures to improve visualization of small values at early time points. *N* = 4–7. *P < 0.05, **P < 0.001. 4%, PLCL_4%A2P_; 8%, PLCL_8%A2P_; PP, polypropylene; SS, sham surgery.

Total collagen, dense collagen and adipose tissue areas at the implantation site were quantified from MTRI-stained B- and C-tissue sections using QuPath’s pixel classification. Percentages of total collagen and adipose tissue relative to the analysis area, and dense collagen relative to total collagen, were also calculated. The average results for the B and C sections of each animal are presented in Figure 9.

PLCL-based groups showed a gradual increase in collagen. At 1 week, the PLCL group had the highest total collagen area (2.9 ± 0.5 mm^2^), being significantly higher than the SS (1.7 ± 0.4 mm^2^; *P *= 0.002), PP (2.3 ± 1.2 mm^2^; *P *= 0.025) and PLCL_8%A2P_ (1.9 ± 0.6 mm^2^; *P *= 0.008) groups. By 1 month, all material nearly doubled their collagen area: PLCL (4.7 ± 0.9 mm^2^), PLCL_4%A2P_ (4.4 ± 1.1 mm^2^), PLCL_8%A2P_ (4.7 ± 1.3 mm^2^) and PP (4.5 ± 1.2 mm^2^). At 6 months, the PLCL_4%A2P_ group had the highest collagen area (6.5 ± 1.6 mm^2^), followed by the PLCL_8%A2P_ (6.1 ± 1.2 mm^2^) group. Both were significantly higher than the SS (2.7 ± 1.2 mm^2^; *P *< 0.01) and PP (3.5 ± 1.5 mm^2^; *P *< 0.05) groups. Collagen percentages at 1 week and 1 month time points were relatively consistent across groups, with the SS group showing slightly higher percentages. By 6 months, the PLCL_4%A2P_ group had a significantly higher collagen percentage (60 ± 9%) compared to the SS group (51 ± 5%; *P *= 0.046).

Dense collagen remained minimal in all groups during the first month. By 6 months, the PLCL_4%A2P_ group had a higher area of dense collagen (0.9 ± 0.3 mm^2^) than SS (0.2 ± 0.2 mm^2^; *P *< 0.001) and PP (0.4 ± 0.4 mm^2^; *P *= 0.04) groups. The SS group also had a smaller dense collagen area than the PLCL (0.7 ± 0.3 mm^2^; *P *= 0.016) and PLCL_8%A2P_ (0.7 ± 0.3 mm^2^; *P *= 0.006) groups. Dense collagen percentage from the total collagen was minimal (2–4%) across all groups during the first month, but increased significantly by 6 months, being lowest in the SS group (5 ± 3%; *P *< 0.05): PP (11 ± 6%), PLCL (14 ± 5%), PLCL_4%A2P_ (15 ± 2%) and PLCL_8%A2P_ (13 ± 3%).

The adipose tissue area remained stable during the first month (0.2 mm^2^) but increased significantly by 6 months: SS (1.9 ± 1.5 mm^2^), PP (1.3 ± 0.7 mm^2^), PLCL (2.2 ± 0.6 mm^2^), PLCL_4%A2P_ (2 ± 1.4 mm^2^) and PLCL_8%A2P_ (2.7 ± 1.6 mm^2^). Adipose tissue percentage was lowest in the PLCL_4%A2P_ group (16 ± 8%) and highest in the SS group (31 ± 8%), with 21 ± 6% for PLCL, 22 ± 9% for PLCL_8%A2P_ and 22 ± 7% for the PP group.

In addition to morphometric analysis, collagenous capsule formation at 6 months was visually graded (Table 3). Overall, the capsule formation was similar among all groups, and no extensive fibrous capsule formation was detected in any material group. However, small unorganized fibrotic clusters (100–400 µm in diameter) were observed in five out of seven animals in the PP group at 6 months (Supplementary material S4).

**Table 3.** Grading of collagenous capsule formation at 6-month time point in blinded Masson trichrome-stained tissue sections (*N* = 5–7)

Grading of dense collagen capsule, *N* (%)						
		PP	PLCL	4%	8%	*P*-value
6 months						ns
	None	0	0 (0)	0 (0)	0 (0)	
	Minimal	4 (57)	2 (40)	4 (57)	2 (29)	
	Moderate	2 (29)	3 (60)	2 (29)	4 (57)	
	Marked	1 (14)	0 (0)	1 (14)	1 (14)	
	Extensive	0 (0)	0	0 (0)	0 (0)	
Total (N)		7	5	7	7	

4%, PLCL_4%A2P_; 8%, PLCL_8%A2P_; PP, polypropylene.

### Immunohistochemical analysis of COL I and COL III deposition in regenerating tissue

**Figure 10.**
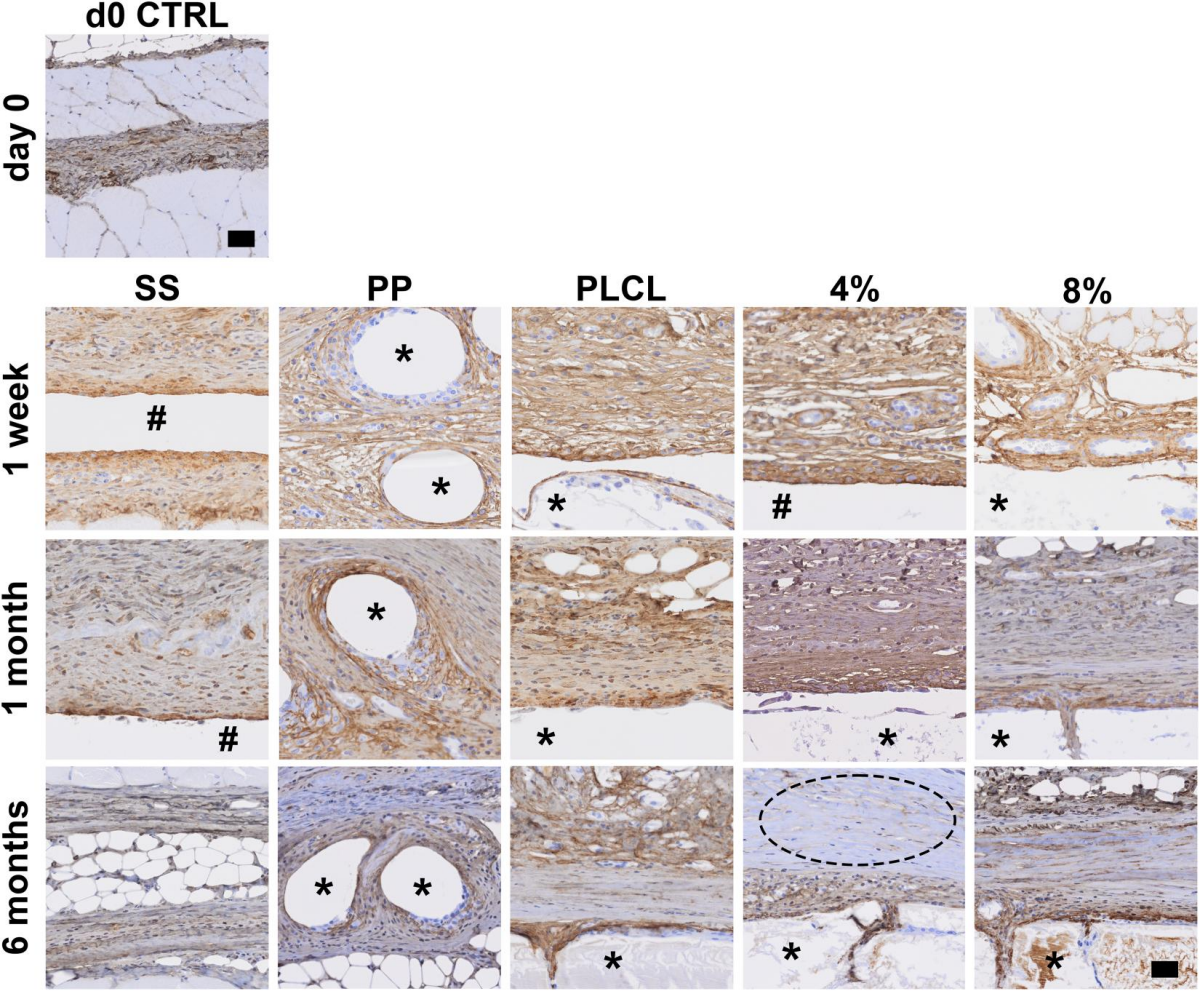
Staining of COL I at time points. Dashed line shows dense collagen region without COL I staining. Scale bar 40 µm. *Biomaterial, #fluid cavity. 4%, PLCL_4%A2P_; 8%, PLCL_8%A2P_; PP, polypropylene; SS, sham surgery.

Histology samples were stained for COL I and COL III to specify the collagen type deposited in the newly formed connective tissue (Figure 10 and Supplementary material S5). However, despite our best efforts, we were not able to get the collagen antibodies to stain the most densely packed regions of fibrous collagen. In general, COL I staining appeared qualitatively similar across all material groups. The staining was most intense in the regions with macrophages near the material surfaces and tissue strands growing into degrading PLCL, PLCL_4%A2P_ and PLCL_8%A2P_ materials. Notably, as the degradation of PLCL, PLCL_4%A2P_ and PLCL_8%A2P_ progressed, more pronounced COL I staining was observed within the fractures of the degrading materials. In addition, in the SS 1-week and 1-month samples, the cell border of the fluid-filled cavities exhibited strong COL I staining. COL III staining was less abundant than COL I and generally observed in newly formed immature loose connective tissue, especially in PLCL, PLCL_4%A2P_ and PLCL_8%A2P_ groups at the 1-month, and in large material fractures at the 6-month time point (Supplementary material S5).

## Discussion

The development of safer and more effective biomaterials for POP repair remains an ongoing challenge. This study is the first to assess the *in vivo* response of absorbable A2P-releasing PLCL_A2P_ membranes, following our previous promising *in vitro* findings [25]. We assessed the effect of A2P-releasing PLCL membranes on tissue strength and collagen formation in a rat abdominal fascia model, focusing on how PLCL, PLCL_4%A2P_ and PLCL_8%A2P_ membranes affect tissue regeneration compared to nonabsorbable PP mesh, particularly evaluating A2P’s effect on *de novo* collagen synthesis.

An optimal biomaterial for POP repair should mimic native pelvic tissue mechanical properties, promote tissue regeneration and degrade at a rate that enables tissue integration without premature loss of support. Importantly, it should enhance tissue strength without excessive stiffness [44]. We have previously demonstrated *in vitro* that A2P increases PLCL membrane hydrophilicity [25], which promotes scaffold degradation rate [25, 40, 45]. In the current study, PLCL_4%A2P_ and PLCL_8%A2P_ membranes showed more advanced degradation and tissue ingrowth compared to plain PLCL as demonstrated by both micro-CT and histological analysis, being more pronounced with higher A2P content. Therefore, A2P appears to enhance *in vivo* tissue integration, which is particularly relevant for POP application, where improved integration might reduce biomaterial-related problems [46, 47].

Although our earlier *in vitro* work indicated that A2P may initially weaken the tensile properties of PLCL [25], both the PLCL_4%A2P_ and PLCL_8%A2P_ groups showed similar or slightly improved tissue tensile properties *in vivo* compared to the plain PLCL group, despite accelerated material degradation. The newly regenerated tissue in the PLCL, PLCL_4%A2P_ and PLCL_8%A2P_ groups compensated for the mechanical support lost due to the material degradation without promoting tissue stiffness. In contrast, the PP group showed increased tissue stiffness over time, consistent with earlier *in vivo* studies [29, 48–50] and further supporting the concerns regarding PP use in POP surgery, where increased tissue stiffness has been associated with the clinical complications of transvaginal PP meshes [51, 52]. Tensile strength of 0.42–0.79 MPa and 17% maximum elongation have been reported for non-prolapsed vaginal tissue [13, 53]. Our results suggest that PLCL, PLCL_4%A2P_ and PLCL_8%A2P_ membranes maintained sufficient structural support during degradation to enable tissue regeneration, highlighting their potential to strengthen connective tissue. Extended observation periods may reveal more about the long-term tissue regeneration following PLCL_A2P_ implantation. For example, a previous *in vivo* study on polylactic acid (PLA)-based meshes reported variable outcomes, with tissue strength declining progressively over 6 months [54] or increasing tissue strength during the first 6 months followed by a significant decrease at 12 months [55].

In the current study, we used an onlay biomaterial implantation model. Therefore, the results cannot be directly compared to the previous studies utilizing abdominal defect models. Additionally, variability in analysis techniques across studies further complicates direct comparisons. Nevertheless, previous studies support the regenerative potential of absorbable materials. For example, electrospun PLCL/porcine fibrinogen patches studied in rat full-thickness abdominal wall defect reconstruction demonstrated complete degradation by 180 days, with the material being replaced by regenerated muscle tissue and collagen [56]. Similarly, in a canine abdominal defect model, PLCL/fibrinogen meshes outperformed PP mesh controls, and the regenerated tissue compensated for the degraded mesh, demonstrating clinical potential, as supported by a 6-year follow-up study for POP [29, 57]. Similarly, in our onlay model, PLCL, PLCL_4%A2P_ and PLCL_8%A2P_ membranes promoted progressive tissue integration and increased tissue strength over time.

As increased collagen deposition is a key indicator of proliferative and remodelling phase in normal tissue healing [58], we analysed the effects on collagen deposition. We detected approximately 60% collagen already at 1 month for both PLCL_4%A2P_ and PLCL_8%A2P_ groups. In comparison to previous studies, a subcutaneous polyamide mesh implant in a rat model showed approximately 25% collagen at 1 month, stabilizing at 60% by 2 months [59], and a polycaprolactone (PCL) mesh implant coated with connective tissue growth factor and rat mesenchymal stem cells reached 50% collagen at 6 months [60]. Therefore, PLCL_A2P_ membranes demonstrate potential for increasing collagen production *in vivo*, as they are of non-animal origin and do not require the use of growth factors or cellular components, for example. Overall, our findings support that PLCL_A2P_ membranes enhance collagen production *in vivo*, consistent with our previous *in vitro* results suggesting that A2P release may promote cellular activity and ECM deposition to improve tissue strength [23, 25, 26]. Notably, as rats can synthetize AA, the observed effects of A2P release may be even more pronounced in potential outcomes for human applications [61].

Both PLCL_4%A2P_ and PLCL_8%A2P_ groups showed nearly twice the collagen area compared to the PP group by 6 months. Importantly, PLCL-based biomaterials did not induce pathological fibrosis, consistent with previous *in vivo* findings [62]. Moreover, excessive fibrosis was not observed in any study group, although all groups had increased dense collagen area at 6 months. Interestingly, despite its smaller surface area, the PP group showed a dense collagen area similar to the PLCL-based groups. The observed dense collagen may either be remodelled into normal tissue over time or progress towards pathological scarring [63]. However, the time points of this study are insufficient to determine the ultimate outcome of the dense collagen. These observations align with the natural foreign body response, where a fibrous capsule forms around foreign materials, which may be resolved during successful biomaterial integration [64].

To our knowledge, this study is the first to report enhanced *in vivo* collagen deposition using A2P-releasing scaffolds in soft tissue applications, whereas previous *in vivo* studies on AA-embedded scaffolds have demonstrated similar effects in bone tissue [45]. We tested 4% and 8% A2P to evaluate the optimal concentrations for PLCL_A2P_ membranes. Generally, PLCL_4%A2P_ appeared to show more favourable results than PLCL_8%A2P_, although differences were not statistically significant. This aligns with previous research, where 5% A2P in a PLA/PCL/gelatin porous scaffold was more effective than 1% and 10% A2P in promoting bone formation in rats [45]. While no cytotoxic effects were detected with 8% A2P in the current *in vivo* study or our previous *in vitro* studies [23, 25, 26], higher concentrations of AA and A2P have been associated with cytotoxicity *in vitro* in previous research [65, 66]. Additionally, a prior study on orally supplemented AA in mice suggests a profibrotic effect of AA in muscle regeneration [67], raising questions on the long-term remodelling of the dense collagen deposition observed in the current study.

We observed stronger COL I staining, particularly in tissue strands growing into material fractures, suggesting that PLCL degradation may promote tissue remodelling, possibly through mechanical stress-induced fibroblast activation or improved cell adhesion [18, 68]. Degradation rate affects tissue regeneration, where too rapid degradation may hinder collagen deposition, while slower degradation may support controlled tissue remodelling [64]. Unfortunately, while we could not characterize collagen types in dense regions, more COL I than COL III staining was generally observed, and COL III was mostly localized in loose, newly formed tissue, particularly in tissue growing through PLCL, PLCL_4%A2P,_ and PLCL_8%A2P_ membranes, indicating active tissue remodelling [58, 69]. These *in vivo* findings are supported by our previous *in vitro* studies, where PLCL_A2P_ membranes increased COL III expression and extracellular COL I production in human vaginal fibroblasts and human adipose-derived stem/stromal cells [23, 25].

Adipose tissue increased in all study groups at 6 months, most likely due to age-related metabolism changes, but it may also possibly be influenced by the surgical procedure. Previous studies have reported adipocyte infiltration at implant sites, potentially driven by local inflammatory responses or mechanical stress [70–72]. However, as adipose tissue naturally increases with age and is normally present in the abdominal fascia, its increase is not necessarily pathological, unlike adipose infiltration in skeletal muscle, which can compromise muscle function [73]. Nevertheless, further research is needed to assess the long-term significance of adipose tissue for PLCL_A2P_ functionality and its implications for POP repair.

While the rat abdominal model is valuable for early-stage biomaterial evaluation due to connective tissue similarities with humans [74], the differences in mechanical load compared to the human pelvic floor limit the direct translation to human conditions. Additionally, this study was conducted using healthy tissue, and the observed effects of A2P may differ in weakened or pathological tissues. Therefore, future studies should assess PLCL_4%A2P_ and PLCL_8%A2P_ membranes in compromised tissues, as well as their long-term regenerative potential in a more physiologically relevant model. Most importantly, as the current study focused on the regenerative potential of the PLCL_4%A2P_ and PLCL_8%A2P_ membranes, a thorough assessment of their elicited inflammatory responses is essential to ensure safety for potential clinical translation. Nevertheless, our findings demonstrate that A2P-releasing PLCL_4%A2P_ and PLCL_8%A2P_ membranes promote collagen deposition and tensile strength, demonstrating promising effects for potential POP applications.

## Conclusions

This study is the first to evaluate the *in vivo* potential of A2P-releasing PLCL membranes. Our findings indicate that PLCL_4%A2P_ and PLCL_8%A2P_ enhance collagen production *in vivo*, while maintaining tissue flexibility, in contrast to PP meshes that appear to promote tissue stiffness. While further studies are required to assess long-term tissue remodelling and elicited inflammatory responses, our results suggest that the bioabsorbable A2P-releasing PLCL membranes could be a promising alternative to traditional PP vaginal meshes for surgical POP repair.

## Supplementary Material

rbaf097_Supplementary_Data
