# Supplementary material for: Ascorbic acid 2-phosphate-releasing poly-l-lactide-co-epsilon-caprolactone membranes enhance tissue regeneration: first in vivo insights for pelvic organ prolapse
Source: Regen Biomater. 2025 Sep 18;12:rbaf097. doi: 10.1093/rb/rbaf097 (PMC12640511; doi:10.1093/rb/rbaf097)
Supplement: rbaf097_Supplementary_Data [file rbaf097_supplementary_data.docx]

# Supplementary Files

**Supplementary S1**

*Quantification of total collagen and dense collagen area*

Total collagen area was measured from the blinded MTRI-stained B- and C- sections using QuPath and its built-in pixel classification tools. A collagen pixel classifier was trained to detect blue-staining collagen, dark-brown nuclei, red-staining muscle tissue, and pink-staining cells. The classifier utilized Random Trees detection at very high resolution (0.55 µm/px) with QuPath’s default settings. First, the collagen pixel classifier was applied to quantify the total collagen area within the analysis area. Next, the collagen area was saved as a detection, which was then used for assessing the approximate amount of dense collagen regions within the total stained collagen. The dense collagen classifier was also based on Random Trees detection, but at a low resolution (4.38 µm/px) with QuPath’s default settings. In the results section, the measured areas of total collagen and dense collagen are reported. In addition, the percentage of total collagen relative to the measured total tissue area in the analysis area and the percentage of dense collagen from total collagen are reported.

*Quantification of adipose tissue area*

The area of adipose tissue within the connective tissue layer was quantified from the blinded MTRI-stained B- and C- sections using a trained pixel classifier in QuPath. The pixel classifier used QuPath’s default settings with Random Trees detection at a very high resolution (0.55 µm/px). It was trained to detect empty spaces within the analysis area, which were then annotated as adipocyte detections (min object size 80 µm^2^ and min hole size 90 µm^2^). After the initial classification, adipocyte detections with circularity < 0.2, solidity < 0.8, and area > 4000 µm^2^ were excluded. Manual adjustments were made to refine exclusions and adipocyte detections. Finally, the areas of individual adipocyte detections were summed to assess the total area of adipose tissue within the analysis area.

**Supplementary S2**

Example images of A2P particles within PLCL_4%A2P_ and PLCL_8%A2P_ membranes as visualized with micro-CT imaging.


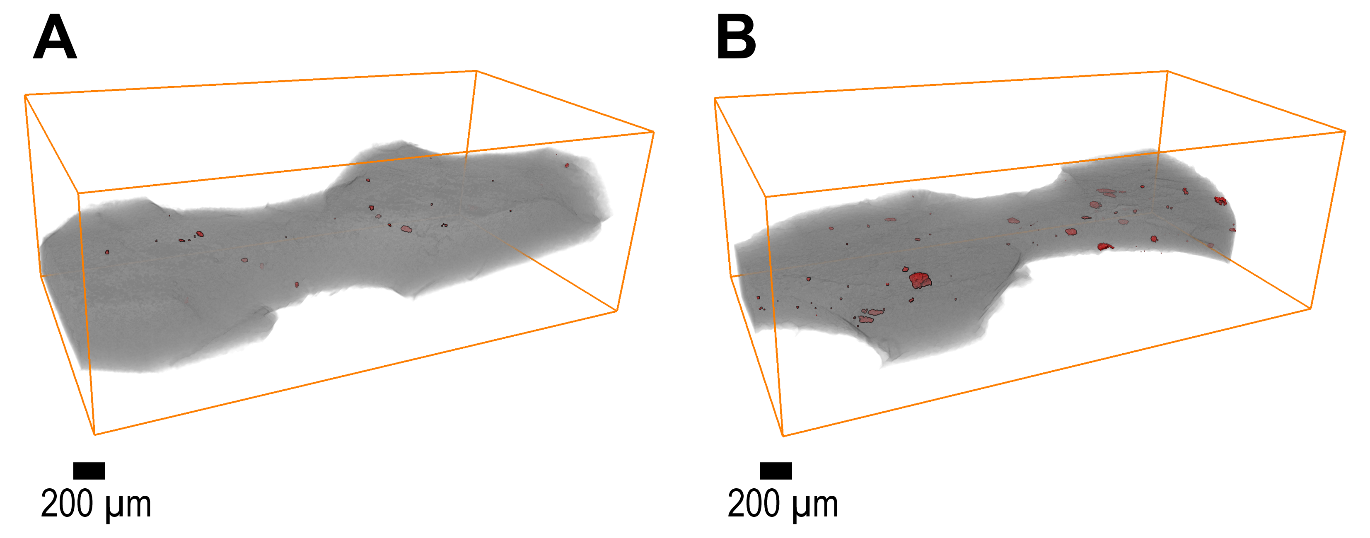


Figure 1 Example images of A2P particles (red) in A) PLCL_4%A2P_ and B) PLCL_8%A2P_ membranes, visualized with micro-CT imaging. The scale bar (200 µm) is for reference only, as calculations were performed on 3D micro-CT data.

**Supplementary S3**

Animal weight change (%) during the observation period.


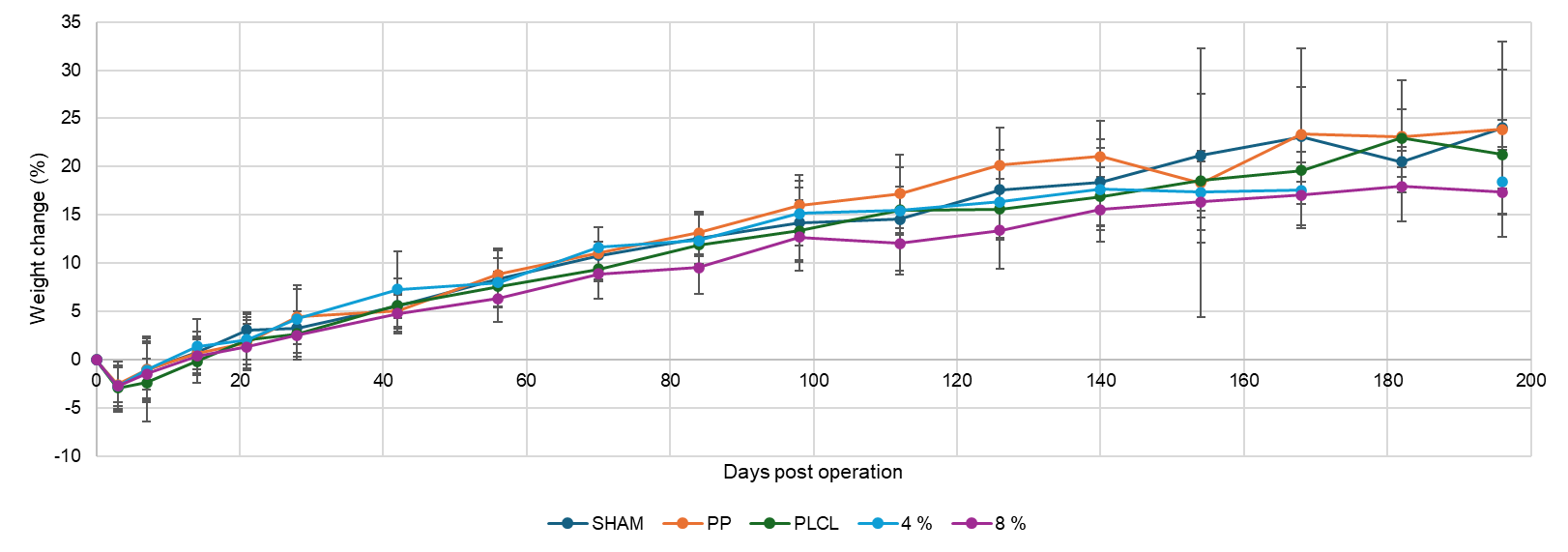


Figure 2 Weight change during the observation period. No statistical differences were detected between groups. SS = Sham surgery, PP = polypropylene, 4% = PLCL_4%A2P_, and 8% = PLCL_8%A2P_. N = 18–21 until 1 week, N = 11–14 between 1 week and 1 month, and N =5–7 after 1 month.

**Supplementary S4**

Observed fibrotic clusters in PP group at 6 months.


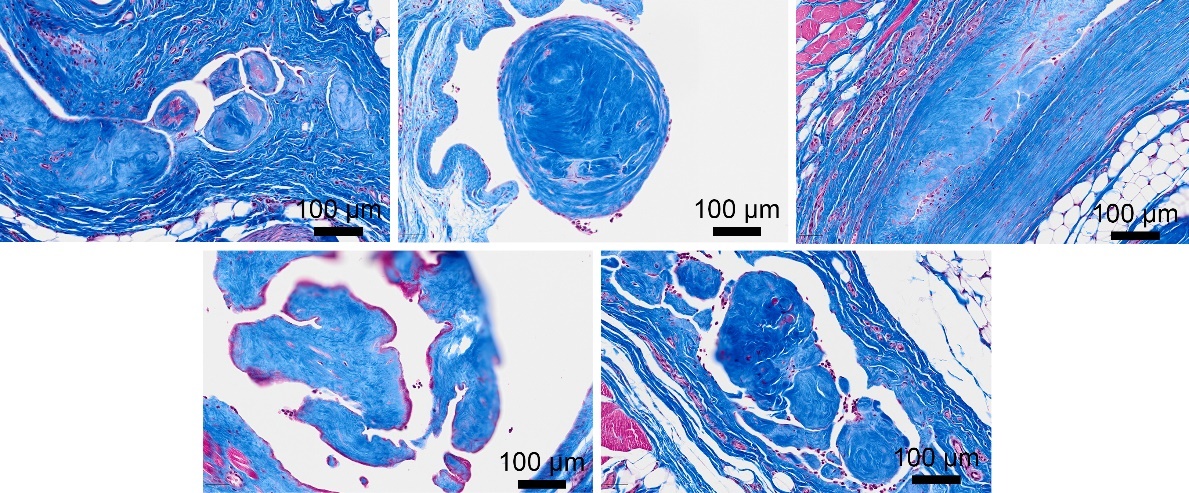


Figure 3 Fibrotic clusters observed in MTRI-stained sections of the PP group at 6 months.

**Supplementary S5**

Staining of COL III at time points.


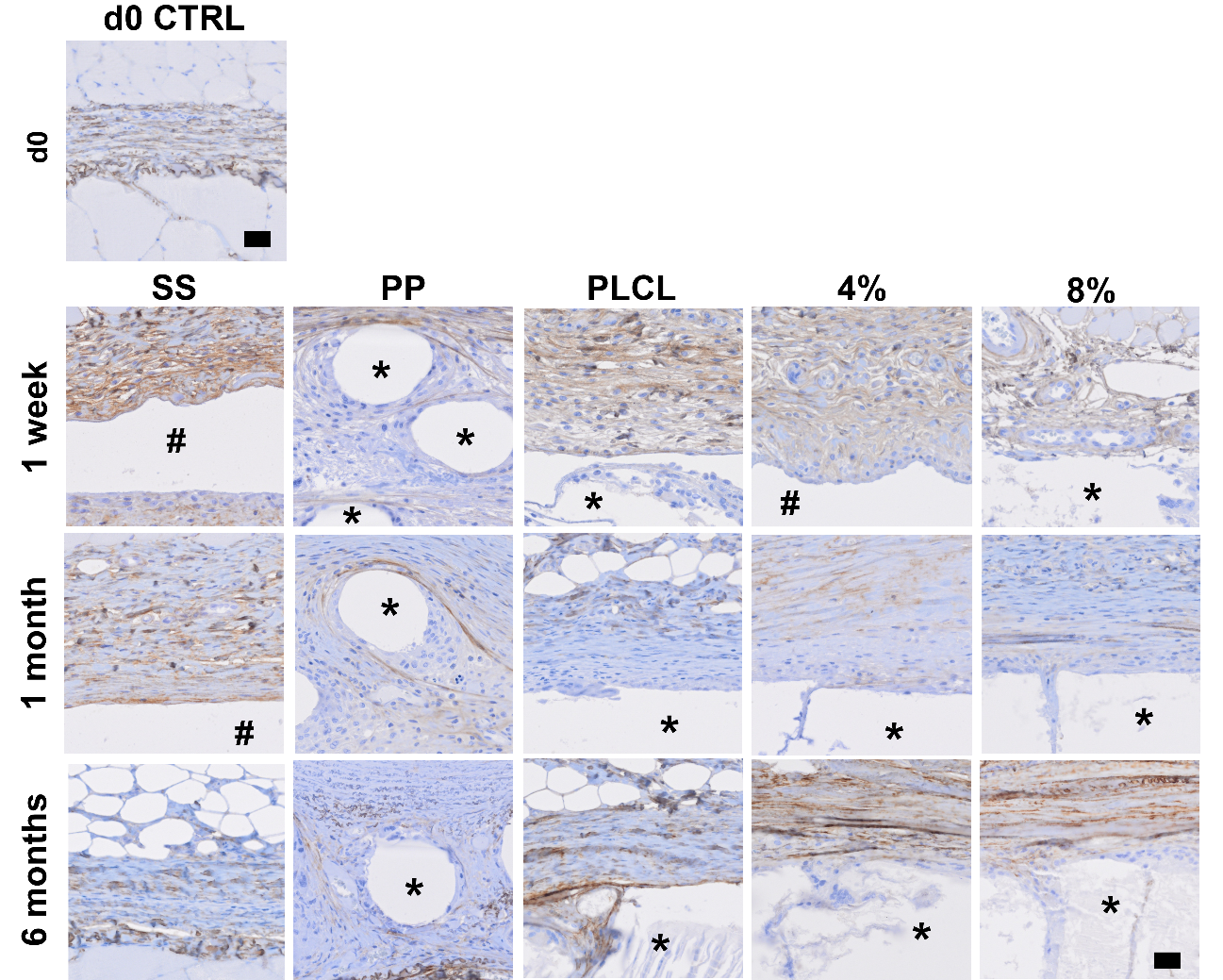


Figure 4 Staining of COL III at time points. SS = Sham surgery, PP = polypropylene, 4% = PLCL_4%A2P_, and 8% = PLCL_8%A2P_. Scale bar = 40 µm. * = material, # = fluid cavity.

Selected images of COL III staining in newly formed tissue.


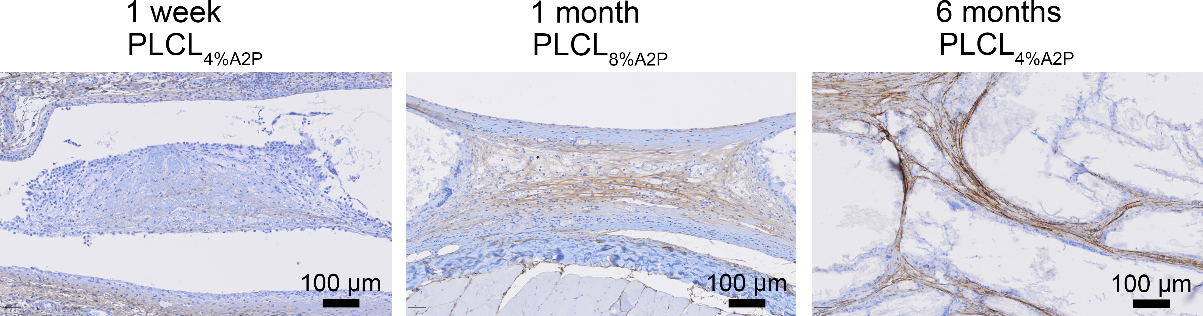


Figure 5 Selected images of COL III staining in newly deposited tissue and in large material fractures.
